# COVID-19 Vaccine Uptake: Prevalence, Health Facility Enablers and Barriers among Adult Tuberculosis Patients across Nairobi County Clinics, Kenya

**DOI:** 10.4236/jtr.2025.131002

**Published:** 2025

**Authors:** Waqo Boru, George Makalliwa, Caroline Musita

**Affiliations:** Department of Environmental Health and Disease Control, Jomo Kenyatta University of Agriculture and Technology, Nairobi, Kenya

**Keywords:** Coronavirus, COVID-19, Enablers, Barriers, Tuberculosis, Vaccine Uptake

## Abstract

Patients with tuberculosis (TB) are at increased risk of developing severe forms of novel coronavirus 2019 (COVID-19), and COVID-19 coinfection aggravates active TB progression through immunosuppression. Despite the availability of COVID-19 vaccines to prevent and reduce COVID-19 transmission, COVID-19 vaccine uptake levels remain low worldwide. In Kenya, an African country with high TB prevalence and TB-HIV coinfection, 47% of the population had received the COVID-19 vaccine by 2023. This study determined health-system enablers and barriers to the COVID-19 vaccine among adult TB patients across Nairobi County TB clinics in Kenya. An analytical cross-sectional study was used. Three hundred eighty-eight TB patients from six TB clinics across six sub-counties in Nairobi were recruited. The participants completed the study questionnaire. After confounding for age, employment status perceived COVID-19 susceptibility, and perceived COVID-19 seriousness, health system enablers of COVID-19 vaccine uptake were consistent and positive messaging on COVID-19 (adjusted odds ratio (aOR) = 4.498; 95% CI: 1.953 - 10.36, p < 0.001), the feeling that the vaccination had significant social benefits was (aOR = 2.632; 95% CI: 1.108 - 6.257, p = 0.028), and enough public awareness about the vaccine (aOR = 2.619; 95% CI: 1.099 - 6.239, p = 0.03). A significant barrier was vaccine preference (aOR = 0.387, 95% CI: 0.179 - 0.838, p = 0.016). This study confirmed health facility factors that enable and hinder COVID-19 vaccine uptake among TB patients. We recommend policy actions to improve TB clinics’ infrastructure and resources to support the enablers and address the barriers.

## 1 Introduction

Since 2020, the coronavirus 2019 (COVID-19) disease has joined tuberculosis (TB) as a leading cause of death around the world [1]. Reports have estimated the COVID-19-related death toll to increase by 20% in countries highly burdened by TB [2]. Infection with COVID-19 warrants excellent attention among the TB population as it causes immunosuppression, especially when TB coexists with other chronic conditions such as HIV. In addition to worsened COVID-19 outcomes, the suppressed immunity aggravates active TB progression [3]. COVID-19 is reported to have derailed efforts against TB by at least five years, with TB prevalence rising by 3.6% and TB treatments reducing by 15% between 2020 and 2021 [4] [5].

Vaccination against COVID-19 minimizes the risk of infection among individuals with TB, minimizing adverse health outcomes. The World Health Organization (WHO), through the Strategic Advisory Group of Experts on Immunization, recommended that all high-risk persons receive a primary vaccine series and an additional booster dose [6]. Countries worldwide have not achieved this recommendation because vaccine uptake levels remain low due to high vaccine hesitancy and other factors. Global primary vaccine series uptake was 62.8% in October 2023, with only 28.01% having received the booster dose [6]. In Africa, where 25% of the global COVID-19 burden was found, only 32.7% of the population had received the primary dose [7] [8]. In Kenya, only 20.6% had received the COVID-19 vaccine by 2022 [9]. Alarmingly, TB incidence in Kenya is 558 per 100,000 persons, with most cases reported in Nairobi county [10]. With 51.5% of the Nairobi population not vaccinated against COVID-19, TB patients are at increased risk of COVID-19 coinfection [11]. Efforts are needed to ensure that all TB patients receive the primary and booster doses to prevent adverse outcomes. Unfortunately, the prevalence of COVID-19 vaccine uptake among TB patients in Nairobi County is unknown.

Many factors hinder COVID-19 vaccine uptake, resulting in low uptake prevalence [12]-[14]. In Kenya, studies identified individual factors such as young age, male gender, lack of decision autonomy in women, perceived health status, herbal medicine beliefs, medical mistrust, myths, religious and cultural factors, fear of side effects and unknown, and lack of understanding as barriers to COVID-19 vaccine uptake [15] [16]. Additionally, factors at the health facility level, such as resource availability, distance to the facility, and vaccination procedures, can significantly affect uptake levels [17]. However, limited data and evidence among TB patients in Kenya hinders further inquiry. No study has been done to identify health system factors influencing COVID-19 vaccine uptake in Kenya. This study contextualized health-facility level factors affecting COVID-19 vaccine uptake in Nairobi County, Kenya. The specific aims of the study were to determine the prevalence of COVID-19 vaccine uptake among TB patients and health-facility level factors that affect uptake within TB clinics in Nairobi County, Kenya.

## 2 Methods

### 2.1 Study Design

This study used an analytical cross-sectional design. Patients were recruited from TB clinics between April and October 2023. The clinics were located within subcounties in Nairobi County, Kenya.

### 2.2 Study Area

The study was carried out in Nairobi County, the capital of Kenya. Nearly four and a half million people live in the area. It has the highest TB burden and nearly 80% of all COVID-19 cases in the country.

#### 2.3 Study Population

Adult patients with confirmed TB who had received appropriate treatment between April and October 2023 were enrolled in the study. Only TB patients over 18 years old and treated in TB clinics were considered. Patients with TB diagnosis, those unwell at the time of the study, and those who could not tell their vaccination status were excluded.

### 2.4 Sample Size Determination

The sample size was determined using Fischer’s formula [18] with an estimated 50% prevalence of COVID-19 vaccine uptake among TB patients in Nairobi. The calculated sample size was then corrected using the finite population correction.

$$n_{0}=\frac{Z^{2}/pq}{e^{2}}$$

*N*_0_ = sample size, *Z* = 1.96, *p* = 0.5, *q* = 1 − *p*, and *e* = 0.05 (95% confidence interval).

$$n_{o}=\frac{\left(1.96^{2}\right)(0.5)(0.5)}{0.05^{2}}=384$$

Finite population correction

$$n=\frac{n_{0}}{1+\frac{\left(n_{0}-1\right)}{N}}$$

where *n* = new sample size, *n*_0_ = estimated sample size, *N* = 4369 TB cases in Nairobi).

$$n=\frac{384}{1+\frac{\left(384-1\right)}{4369}}=353$$

This was adjusted upward by 10% (35) covering for non-response, therefore, the final sample size for the study was 388 respondents

N=388

### 2.5 Sampling

Probability to proportion sampling (PPS) and purposive sampling were used to select the six largest TB clinics with high TB patient numbers across six sub-counties from more than 1000 registered health facilities in Nairobi County. These sampling techniques led to the selection of St. Mary’s Mission Hospital, Riruta Health Centre, Rhodes Chest Clinic, Mama Lucy Kibaki Hospital, MMM Mukuru Health Centre, and Kahawa West Health Centre. TB patients were selected at random from these facilities.

### 2.6 Data Collection Instrument

Data on social-demographic, vaccination status, and health-facility level factors was collected using a semi-structured questionnaire developed by the research team. Its reliability and validity were tested in a pilot study using twelve TB patients in a level IV hospital in the neighboring county of Kiambu. The pilot test found a high reliability of 0.88, but it was revised to reorder the questions and remove ambiguous words. The questionnaire was uploaded to a digital Application called Kobo Collect, which the research assistants could access.

### 2.7 Data Collection

Eight trained research assistants collected data from the six facilities. Due to the large volume of TB patients, two assistants collected data at Mama Lucy Kibaki Hospital and Rhodes Chest Clinic. In contrast, one assistant collected data at the other four hospitals. The research assistants were given a tablet with the Kobo Collect App to access the questionnaire. They contacted TB patients visiting the clinics to explain the study. Patients willing to participate signed a consent form before responding to the questionnaire. The research assistant entered the responses in the application as provided by the participants. The data was then exported from the app as an Excel file.

### 2.8 Data Analysis Plan

Categorical data was analyzed in frequency and percentage and presented in tables. Mean and standard deviation (SD) summarized continuous data. Bivariate and multivariate logistic regression models determined the association between COVID-19 vaccine uptake and health-facility level factors. Associations statistically significant in the bivariate model were entered into the multivariate model after checking for multicollinearity. All analyses were done in Statistical Package for Social Scientists (SPSS) version 26. A p-value of less than 0.05 was statistically significant.

## 3 Results

Data was successfully collected from all six selected TB clinics and a total of 388 participants were engaged in the quantitative study, all completing the questionnaires. The entire *N* = 388, participants’ entries were included in the data analysis and the findings reported herein

### 3.1 Social Demographic Data

Out of the 388 participants, 36.6% were female. Nearly half (48.7%) were between 20 and 35 years, with the mean (SD) age of 37.4 ± 11.6 years. The majority of the participants have secondary education (47.2%), are employed (32.5%), and have a monthly income of less than 20,000 Kenya Shillings (74.7%). Nearly 75% attended government hospitals for TB treatment, and those vaccinated took Astra-Zeneca (Table 1).

### 3.2 Prevalence of COVID-19 Vaccine Uptake

About 46% (179/388) of the participants reported receiving the COVID-19 vaccine at the time of the interview, with 8% (31/388) having partially received the primary vaccine series only. But 54% (209/388) reportedly had not received any vaccine dose/brand (Table 2).

### 3.3 Health Facility Level Factors Influencing COVID-19 Vaccine Uptake

A multicollinearity check was done in the multivariate regression model using Variance Inflation Factor (VIF) and Tolerance values for all variables statistically significant in the bivariate model. The VIF values for all variables were less than 5, indicating a low risk of multicollinearity. All significant variables were included in the multivariate model without the risk of overfitting and inaccurate estimates. The model was adjusted for confounding factors, including age and employment status as sociodemographic factors and mistrust of government vaccine management, perceived COVID-19 susceptibility, and perceived COVID-19 seriousness as individual-level factors significantly associated with COVID-19 vaccine uptake (Table 3).

After confounding for age, employment status perceived COVID-19 susceptibility, and perceived COVID-19 seriousness, consistent and positive messaging on COVID-19 was four and half times more likely to influence TB patients to be vaccinated (adjusted odds ratio (aOR) = 4.498; 95% CI: 1.953 - 10.36, p < 0.001). The feeling that the vaccination had significant social benefits was nearly three times more likely to influence vaccination (aOR =2.632; 95% CI: 1.108 - 6.257, p = 0.028), while having enough public awareness about the vaccine was nearly three times more likely to influence participants vaccination (aOR = 2.619; 95% CI: 1.099 - 6.239, p = 0.03). A vaccine preference significantly hindered vaccination by nearly 61% (aOR) = 0.387, 95% CI: 0.179 - 0.838, p = 0.016). All the other factors did not have a statistically significant association based on the statistically significant value associated with the adjusted odds ratio (Table 4).

## 4 Discussion

Developing a COVID-19 infection in TB patients is detrimental and deadly. Generally, COVID-19 vaccines could prevent the infection, but vaccine uptake remains low due to various factors, including health-system factors [12]-[14]. In Kenya, the prevalence of COVID-19 vaccine uptake among TB patients. Nairobi County in Kenya, which is the epicenter of COVID-19 infection and has the highest TB burden in the country, was the study area for our study. The study determined the prevalence of COVID-19 and explored health system-level factors influencing vaccine uptake among TB patients attending selected TB clinics in Nairobi County, Kenya.

### 4.1 Prevalence of COVID-19 Vaccine Uptake

Our study found that less than 40% of the adult TB patients reported receiving the full COVID-19 vaccination dose. These findings do not align with Palupi *et al*. [19], who reported that 58.8% of adult TB patients had received the COVID-19 vaccine. They are also inconsistent with Wang *et al*. [20], who reported that 68.6% of TB/COVID-19 coinfected patients had received at least two doses of the COVID-19 vaccine. Our study’s prevalence of COVID-19 vaccine uptake was low because of various health facility-level factors typically found in developing countries. Making informed policy recommendations requires understanding these factors and their level of statistical and clinical significance in influencing COVID-19 vaccine uptake. These health system-level factors are discussed below.

### 4.2 Health Facility Level Factors Influencing COVID-19 Vaccine Up-Take

The study findings revealed that health facility-level factors that positively influenced COVID-19 vaccine uptake relate to communication and awareness about the COVID-19 vaccine. Specifically, the findings showed that having public awareness, consistency, and accuracy of safety messages, as well as vaccination having social benefits of the COVID-19 vaccines, statistically significantly influenced vaccine uptake. Most adult TB patients who thought consistent and accurate safety messages were influential were vaccinated, compared to most adult TB patients who thought these messages had no influence. The 95% confidence interval for the consistent and accurate safety message variable was 1.953 and 10.36, indicating a less precise estimate that receiving consistent and accurate safety messages about the COVID-19 vaccine significantly influenced vaccination by increasing the odds of vaccine uptake nearly 4.5 times. The World Health Organization [WHO] emphasizes the importance of accurate vaccine safety communication in empowering people to make informed, evidence-based decisions about uptake [21]. These findings agree with those of Rajshekar *et al*., who reported that assurance of vaccine safety through accurate communication promoted uptake of the COVID-19 vaccine and reduced vaccine hesitancy [22]. They also agree with Bono *et al*., who reported a lack of adequate health messaging as the main barrier to vaccine uptake in low- and middle-income countries [23]. Bono *et al*. found that lack of education on side effects was associated with a 41.2% hindrance to uptake [23].

The current study found that patients who felt that COVID-19 vaccination had social benefits were more likely to be vaccinated. The 95% confidence interval for the emphases on the social benefits variable was 1.108 and 6.239, indicating a less precise estimate that emphasizing the social benefits of the COVID-19 vaccine significantly influenced vaccination by increasing the odds of vaccine uptake 2.6 times. Emphasizing the social benefits creates reputational concerns, including social pressure to get vaccinated. In other words, those not vaccinated are moralized through suggestions that people are all linked together as a social entity, and failure to vaccinate puts other people, including families and friends, at increased risk. These findings are consistent with James *et al*., who reported that persuasive messages showing the social benefits of the COVID-19 vaccine increase uptake intentions [24].

The clinical significance of the findings on the two enablers of COVID-19 vaccine uptake relates to the positive impact on public health by implementing interventions that leverage community-based messaging to increase vaccination rates among high-risk groups such as TB patients. They also relate to influencing behavior by framing vaccine uptake as a social responsibility and aligning it with behavioral theories such as the health belief model. The practical implications of the findings include implementing community-based health campaigns that focus on messaging and accurate information sharing, emphasizing social benefits. These campaigns should use trusted health workers and community leaders to promote vaccine uptake. Health clinics should also establish peer support programs where vaccinated TB patients can share their experiences with those unvaccinated to influence their attitude and perception of vaccine uptake. The peer support program should leverage social media and community groups to reinforce the messaging. Furthermore, TB clinics should integrate targeted vaccine education, emphasizing the social benefits of vaccination.

COVID-19 vaccine preference negatively influenced COVID-19 vaccination. The 95% confidence interval for the having preferred vaccines variable was 0.179 and 0.838, indicating a precise estimate that having vaccine preference significantly influenced vaccination by lowering vaccine uptake by over sixty percent. Perceived patient preferences on specific vaccines based on recommendations by their health providers or family members or personal experience on people receiving them can influence uptake.

The clinical significance of the findings on vaccine preference as a barrier to COVID-19 vaccine uptake includes challenges with COVID-19 control and the need to address misconceptions. There is an increased likelihood that TB patients who insist on specific vaccines may delay or refuse to take the COVID-19 vaccine, increasing the risks of complications. Preference to specific COVID-19 vaccines may prevent achieving herd immunity, which might affect health outcomes for vulnerable groups. There is a need to address the misconception that specific vaccines are more effective and safer than others through education on the equal safety and effectiveness of different COVID-19 vaccines. The practical implications include making multiple COVID-19 vaccines available to patients. Clinics with vaccine varieties are more likely to have TB patients receive the COVID-19 vaccine because of the availability of the preferred vaccine. According to Abraham *et al*., the availability of various vaccines to satisfy client preferences facilitates COVID-19 vaccine uptake [25].

### 4.3 Study Strengths and Limitations

A key study strength is that it used a multivariate regression model that allowed it to evaluate the influence of multiple independent variables on vaccine uptake while adjusting for confounders. Additionally, regression provides adjusted odds ratios (ORs) or coefficients, giving a clearer understanding of the strength and direction of associations while accounting for other variables. One limitation of the study was that it used a cross-sectional design that limited the findings, particularly the odds ratio, to only show association but not the cause-effect relationship [26]. Another limitation was that the study was based on self-reported data, and the accuracy of results depended on participants’ trustworthiness. Self-reported data is prone to recall, social desirability, reporting/response, and acquiescence biases that might contribute to inaccurate estimates of COVID-19 vaccine uptake and affect the conclusions about the influence of health-facility factors.

## 5 Conclusion

Infection with COVID-19 as an individual with TB aggravates active TB and worsens COVID-19 health outcomes. Uptake of the COVID-19 vaccine could prevent the effects, but it’s influenced by many factors, including those related to health facilities where TB patients receive treatment. Our study revealed that COVID-19 vaccine uptake among TB patients remains low in Kenya, particularly in Nairobi County. Health facility-level factors positively influencing COVID-19 vaccine up-take are related to supply chain, communication, and awareness, while registration requirements before vaccination hindered vaccine uptake. Healthcare facilities with various vaccines, those providing consistent and accurate safety messages and emphasizing the social benefits of vaccination, increased COVID-19 vaccine uptake.

## 6 Recommendations

We recommend that TB clinics sustain communication and awareness of the severity of respiratory infection to the TB population and the importance of accelerating COVID-19 vaccination uptake. There is also needed to increase public trust and confidence around the COVID-19 vaccine through positive messaging to demystify misinformation. Emphasize on data privacy & protection (registration) for accountability and documentation purposes. Finally, future studies should utilize designs that investigate cause-effect relationships between the health-system factors and COVID-19 vaccine uptake.

## Figures and Tables

**Table 1:** Social-demographic characteristics.

	Sociodemographic	*n*	%
Age	<20	7	1.8
	20 - 35	189	48.7
	36 - 50	135	34.8
	51 - 65	51	13.1
	> 65	6	1.5
Gender	Female	142	36.6
	Male	246	63.4
Marital stats	Single	135	34.8
	Separated/Widowed	41	10.6
	Married	212	54.6
Education	None	16	4.1
	Primary	102	26.3
	Secondary	183	47.2
	Tertiary	87	22.4
Occupation	Unemployed	142	36.6
	Self	120	30.9
	Formal	126	32.5
Monthly Income	≥ 20,000	290	74.7
	20,001 - 50,000	70	18.0
	50,001 - 100,000	28	7.2
	>100,000	0	0.0
TB Clinic Type	Government	289	74.5
	Private	57	14.7
	Faith-Based	42	10.8
Vaccine received	AstraZeneca	81	45.3
	Johnson& Johnson	49	27.4
	Moderna	16	8.9
	Pfizer	32	17.8
	Sinopharm	1	0.6

Note. Mean age = 22.8 (SD = 12.4) years.

**Table 2:** Prevalence of COVID-19 vaccine uptake and type of vaccine.

Variables	Sociodemographic characteristics		
	Categories	Frequency (N)	Percent (%)
Vaccine stats	Fully vaccinated	148	38.1
	Partially vaccinated	31	8.0
	Not vaccinated	209	53.9

**Table 3:** Sociodemographic and individ**u**al-level factors associated with COVID-19 vaccine uptake.

Variable		Logistic regression			
		Odds ratio (OR) (95% CI)	p-value	Adjusted OR (95% CI)	p-value
**Sociodemographic factors**					
Age	>35 Yrs	1.6 (1.077 - 2.406)	0.025	1.891 (1.185 - 3.427)	0.03
	≤35 Yrs	ref			
Gender	Female	1.1 (0.706 - 1.617)	0.833	-	-
	Male	ref			
Marital status	Single/Separated	0.9 (0.588 - 1.307)	0.540	-	-
	Married	ref			
Education level	Primary or None	0.8 (0.520 - 1.243)	0.376	-	-
	Secondary or Higher	ref			
Employment status	Formal/Self Employed	1.9 (1.266 - 2.959)	0.002	2.83 (1.191 - 6.705)	0.018
	Unemployed	ref			
Monthly income	>Ksh. 20,000	1.7 (1.079 - 2.717)	0.026	0.90 (0.359 - 2.270)	0.828
	≤Ksh. 20,000	ref			
Religion	Christians	1.3 (0.701 - 2.422)	0.438	-	-
	Muslims or Other	ref			
**Individual level factors**					
Myths/Misconceptions	Had Influence	0.43 (0.254 - 0.725)	0.002	1.344 (0.471 - 3.839)	0.581
	No Influence	ref			
Stigma on TB and HIV	Had Influence	0.59 (0.358 - 0.983)	0.054	-	-
	No Influence	ref			
Mistrust on Vaccine	Had Influence	0.19 (0.117 - 0.314)	< 0.001	0.820 (0.295 - 2.285)	0.705
	No Influence	ref			
Consider TB a Risk Factor	Had Influence	2.23 (1.418 - 3.498)	0.001	0.587 (0.240 - 1.431)	0.241
	No Influence	ref			
Perceived Covid-19 Susceptibility	Had Influence	8.09 (4.916 - 13.32)	<0.001	2.901 (1.258 - 6.688)	0.012
	No Influence	ref			
Prefer Natural Immunity	Had Influence	0.59 (0.384 - 0.890)	0.014	0.918 (0.389 - 2.165)	0.844
	No Influence	ref			
Perceived Covid-19 Seriousness	Had Influence	11.83 (6.887 - 20.32)	< 0.001	3.294 (1.130 - 9.604)	0.029
	No Influence	ref			

**Table 4:** Health-facility level factors associated with COVID-19 vaccine uptake.

Variable	Logistic regression					
		Odds ratio (OR) (95% CI)	p-value	Adjusted OR (95% CI)	p-value	VIF
Had Preferred Vaccine	Had Influence	0.073 (0.043 - 0.123)	<0.001	0.387 (0.179 - 0.838)	0.016	2.223
	No Influence	ref				
Had a Variety of Vaccines	Had Influence	0.107 (0.067 - 0.172)	<0.001	0.581 (0.267 - 1.264)	0.171	1.969
	No Influence	ref				
Facility/Centre Opening Hours	Had Influence	5.88 (3.712 - 9.329)	<0.001	1.556 (0.668 - 3.623)	0.305	1.903
	No Influence	ref				
Consistent and Accurate Safety Message	Had Influence	22.9 (12.37 - 42.42)	<0.001	4.498 (1.953 - 10.36)	<0.001	2.066
	No Influence	ref				
Emphases on Social Benefits	Had Influence	17.28 (9.506 - 31.40)	<0.001	2.632 (1.108 - 6.257)	0.028	2.208
	No Influence	ref				
Public Awareness of Medication	Had Influence	19.00 (10.30 - 35.07)	<0.001	2.619 (1.099 - 6.239)	0.03	2.278
	No Influence	ref				
Mandatory Vaccination Certificate	Had Influence	5.25 (3.327 - 8.292)	<0.001	0.960 (0.449 - 2.053)	0.916	1.406
	No Influence	ref				
